# The lysyl oxidase like 2/3 enzymatic inhibitor, PXS‐5153A, reduces crosslinks and ameliorates fibrosis

**DOI:** 10.1111/jcmm.14074

**Published:** 2018-12-09

**Authors:** Heidi Schilter, Alison D. Findlay, Lara Perryman, Tin T. Yow, Joshua Moses, Amna Zahoor, Craig I. Turner, Mandar Deodhar, Jonathan S. Foot, Wenbin Zhou, Angelique Greco, Amar Joshi, Benjamin Rayner, Sarah Townsend, Alberto Buson, Wolfgang Jarolimek

**Affiliations:** ^1^ Drug Discovery department Pharmaxis Ltd. Sydney NSW Australia; ^2^ Heart Research Institute Sydney NSW Australia; ^3^ Sydney Medical School University of Sydney Sydney NSW Australia; ^4^ Centre for Liver Research Institute of Immunology National Institute for Health Research Liver Biomedical Research Unit University Hospitals Birmingham UK; ^5^ Birmingham NHS Foundation Trust University of Birmingham Birmingham UK

**Keywords:** collagen crosslinking, fibrosis, LOXL2, LOXL3, lysyl oxidase, PXS‐5153A

## Abstract

Fibrosis is characterized by the excessive deposition of extracellular matrix and crosslinked proteins, in particular collagen and elastin, leading to tissue stiffening and disrupted organ function. Lysyl oxidases are key players during this process, as they initiate collagen crosslinking through the oxidation of the ε‐amino group of lysine or hydroxylysine on collagen side‐chains, which subsequently dimerize to form immature, or trimerize to form mature, collagen crosslinks. The role of LOXL2 in fibrosis and cancer is well documented, however the specific enzymatic function of LOXL2 and LOXL3 during disease is less clear. Herein, we describe the development of PXS‐5153A, a novel mechanism based, fast‐acting, dual LOXL2/LOXL3 inhibitor, which was used to interrogate the role of these enzymes in models of collagen crosslinking and fibrosis. PXS‐5153A dose‐dependently reduced LOXL2‐mediated collagen oxidation and collagen crosslinking in vitro. In two liver fibrosis models, carbon tetrachloride or streptozotocin/high fat diet‐induced, PXS‐5153A reduced disease severity and improved liver function by diminishing collagen content and collagen crosslinks. In myocardial infarction, PXS‐5153A improved cardiac output. Taken together these results demonstrate that, due to their crucial role in collagen crosslinking, inhibition of the enzymatic activities of LOXL2/LOXL3 represents an innovative therapeutic approach for the treatment of fibrosis.

## 1 INTRODUCTION

Fibrosis is a progressive disease characterized by extensive scarring and tissue stiffening, which develop from chronic tissue damage and incessant wound‐healing processes.^1^, ^2^, ^3^ During fibrosis, the excessive deposition, accumulation and crosslinking of collagen causes more lesions to mature, resulting in scarring and, potentially, irreversibility of disease. Fibrosis can develop in nearly any organ and is an important driver of end‐stage organ failure and mortality in a number of chronic diseases.^4^, ^5^ Thus, the development of effective therapeutic avenues that halt or reverse disease progression is urgently required.

In healthy tissues, collagens play a key role by providing strength, stability and integrity.^6^ These properties are based on a highly organized molecular structure in which three collagen chains are interwoven into a triple helix.^6^ In addition, collagen fibrils are further strengthened by covalent crosslinks formed enzymatically between the collagen molecules.^6^, ^7^ This process has been confirmed to be driven by lysyl oxidases, as when enzymatically active LOX was incubated (in vitro) with purified collagen it resulted in dihydroxylysinonorleucine (DHLNL) as well as pyridinoline (PYD) formation.^8^ Specifically, lysyl oxidases oxidatively deaminate lysine and hydroxylysine residues in the telopeptide domains of the collagen molecule to form the corresponding aldehydes (allysines or hydroxyallysines) (Figure S1).^7^, ^9^, ^10^, ^11^ These aldehydes react spontaneously, either with other aldehydes or with unmodified lysine or hydroxylysine residues, thereby resulting in the formation of crosslinks. This process results in dimeric immature crosslinks, such as DHLNL (hydroxyallysine‐derived crosslink) as well as hydroxylysinonorleucine (HLNL) (allysine‐derived crosslink). The reaction of immature crosslinks with an additional aldehyde results in the formation of very stable trimeric, mature crosslinks PYD and deoxypyridinoline (DPD) (hydroxyallysine and allysine‐derived crosslink, respectively). During fibrosis, the disproportionate accumulation of collagen leads to excessive maturation of crosslinks, resulting in the key characteristics of the disease: tissue stiffening, scarring and irreversibility.

Lysyl oxidases constitute a family of copper‐dependent amine oxidases comprised of five members, lysyl oxidase (LOX) and lysyl oxidase‐like 1‐4 (LOXL 1‐4).^2^ They show a high degree of homology in the catalytic carboxy terminal end and more divergence in the rest of the sequence.^12^ Lysyl oxidases regulate many biological processes including extracellular matrix (ECM) stabilization, cellular growth and homeostasis. Their protein expression has been positively correlated with fibrotic diseases in many different tissues including liver, lung and kidney.^13^, ^14^, ^15^, ^16^ In addition, certain members of the family ‐in particular LOX and LOXL2‐ have been widely associated with cancer progression and metastasis.^13^, ^17^, ^18^ Early research with a functional antibody for LOXL2 (AB0023), demonstrated efficacy in various pre‐clinical models of fibrosis and cancer.^13^ Recently, PAT‐1251 a selective small molecule inhibitor of LOXL2 also demonstrated potential as an anti‐fibrotic agent in pre‐clinical models.^19^ Although the primary role of this family of enzymes is ECM remodelling, a number of extra‐ and intracellular functions have also been reported that are independent of the enzymatic activity. Extracellularly, LOXL2 has been shown to signal through β‐integrin in cancer‐associated fibroblasts^20^ and intracellularly, LOXL2 and LOXL3 have been associated with epithelial to mesenchymal transition (EMT).^21^, ^22^, ^23^ Additionally, LOXL2 is a key player for heterochromatin formation via Snail‐dependent mechanisms.^21^, ^22^, ^24^ Moreover, one study showed that LOXL2 is a negative regulator of Notch1 transcription, thereby attenuating epidermal differentiation.^25^

Despite the extensive body of work on lysyl oxidases and the considerable therapeutic potential of LOXL2 and LOXL3 inhibition, no studies have provided unequivocal evidence for the relationship between the enzymatic activities of LOXL2/LOXL3, and the reduction of crosslinks resulting in therapeutic benefits in models of fibrosis. Herein, we set out to develop PXS‐5153A, an innovative small molecule inhibitor with complete LOXL2/LOXL3 enzymatic inhibition ‐unlike the currently available antibody (simtuzimab)‐ with faster onset and higher potency for LOXL2/LOXL3 than the small molecule inhibitor racemate of PAT‐1251.

Using PXS‐5153A, we were able to dissect the enzymatic function of LOXL2 and LOXL3 on the formation of different crosslink subtypes during fibrosis and examine the relevance of LOXL2/LOXL3 inhibition on disease severity as well as organ function recovery.

## 2 MATERIALS AND METHODS

### 2.1 Fluorometric enzymatic activity assays

The measurement of the enzymatic activity of all lysyl oxidase family members was based on the detection of hydrogen peroxide with an Amplex‐Red oxidation assay, as described in Zhou et al^26^ Recombinant human semicarbazide sensitive amine oxidase (SSAO/VAP1), diamine oxidase (DAO) and monoamine oxidases A and B (MAO‐A and MAO‐B) assays were performed as previously described.^27^ For detailed enzymatic procedures, see supporting information.

### 2.2 Off‐target activity

PXS‐5153A was tested at Eurofins Cerep Panlabs Taiwan, Ltd in the “Hit Profiling Screen”, which tested 30 different targets.

### 2.3 Pharmacokinetic studies

Studies were performed by Pharmalegacy (Shanghai, China), GVK (Hyderabad, India) and Sundia, (Shanghai, China), with local ethics approval. Wistar rats were administered PXS‐5153A orally at 10 mg/kg or intravenously at 5 mg/kg, while C57/BL6 mice received PXS‐5153A orally or intravenously at 5 mg/kg. Plasma was analysed for PXS‐5153A by high‐performance liquid chromatography‐mass spectrometry/mass spectrometry (LCMS/MS).

### 2.4 Collagen oxidation assay

Collagen oxidation assay was based on the release of hydrogen peroxide as previously described^27^ and detailed in the supporting information. Briefly, collagen was combined with rhLOXL2 with or without the pan‐lysyl oxidase inhibitor BAPN (β‐aminoproprionitrile, 100 μmol/L, Sigma‐Aldrich) or PXS‐5153A. An Amplex reaction mixture was added into each well. The slope per minute of the kinetic curves for each sample was calculated using MARS data analysis software (BMG labtech) in the linear phase (between the 20 and 40 minute time points).

### 2.5 In vitro crosslinking assay

About 200 μL of 3 mg/mL collagen (rat tail, type I, Thermo Fisher) was combined with 800 μL of 50 mmol/L sodium borate buffer (pH 8.2) and 20 nmol/L of rhLOXL2 (with or without inhibitor, PXS‐5153A 200 nmol/L). Enzyme/inhibitor were replenished daily for 5 days. Samples were incubated at 37°C and crosslinks were extracted on day 7.

### 2.6 Protein, hydroxyproline and collagen crosslinking analysis

About 10mg of freeze‐dried samples were reduced with NaBH_4_. Pellet then underwent acid hydrolysis in 6 mol/L HCl at 100°C for 24 hours. Hydroxyproline and crosslinks were extracted from the hydrolysate using an automated solid phase extraction system (Gilson GX‐271 ASPECA system). After extraction and drying, hydroxyproline and crosslinks were analysed by UHPLC‐ESI‐MS/MS on a Thermo Dionex UHPLC and TSQ Endura triple quad mass spectrometer. Total protein was quantified in the samples using a commercially available kit (QuickZyme Biosciences, Leiden, The Netherlands). For detailed procedures, see supporting information.

### 2.7 CCl_4_‐induced liver fibrosis

The study was performed by Pharmalegacy with approval from local ethics committee. Sprague Dawley rats were orally administered with 0.25 μL/g Carbon tetrachloride (CCl_4_) in olive oil solution, starting from day 0, 3 times per week for 6 weeks. Animals were killed 48 hours after the last CCl_4_ administration. PXS‐5153A was given by oral gavage after 3 weeks of CCl_4_ administration and continued throughout the remainder of the study at 3 mg/kg (low dose) or 10 mg/kg (high dose) once a day or 10 mg/kg (high dose) three times a week. Alanine aminotransferase (ALT) and aspartate aminotransferase (AST) levels were assessed in the plasma. One lobe of the liver tissue was fixed in 10% formalin, stained for Sirius red and the percentage coverage area was measured. The remainder of the liver was snap frozen and used for protein, hydroxyproline and crosslink analysis.

### 2.8 NASH‐induced liver disease

The study was performed by Stelic MC, Inc. (Tokyo, Japan) with approval from local ethics committee. NASH was established in male C57/BL6 mice by a single subcutaneous injection of 200 μg streptozotocin (Sigma‐Aldrich) after birth and with a high fat diet (CLEA Japan) ad libitum after 4 weeks of age (day 28 ± 2) until 14 weeks of age. Mice were orally administered with 10 mg/kg PXS‐5153A once daily from 8 to 14 weeks of age. ALT levels were assessed in the plasma. One lobe of the liver tissue was fixed in 10% formalin, stained for Sirius red and the percentage coverage area was measured; HE staining was performed to estimate non‐alcoholic fatty liver disease (NAFLD) activity score according to the criteria of Kleiner et al^28^ The remainder of the liver was snap frozen and used for protein, hydroxyproline and crosslink analysis.

### 2.9 Myocardial infarction

The study was performed by CL Laboratory (Baltimore, USA) with approval from the Institutional Animal Care and Use Committee. Myocardial infarction (MI) was induced in C57/BL6 mice by occluding the left coronary artery. The same surgery but without occluding the left coronary artery was used as a sham control. At 24 hours post‐surgery, animals received echocardiography. Infarcted mice with high cardiac function (FS > 40%) or low cardiac function (FS < 10%) were excluded from the study. The remaining mice were treated q.d., p.o., with 25 mg/kg of PXS‐5153A for 4 weeks. At the end of the experiment, echocardiography was performed on mice to assess left ventricular function and remodelling, followed by heart collection. The heart was fixed with 10% formalin. Fibrosis was assessed in the non‐infarct area. Fibrotic blue area and whole non‐infarct area were measured using computerized planimetry (*Image J*). The fibrotic area was presented as a percentage of the whole non‐infarct area. Three random fields per heart were counted, averaged and a total of 30‐45 fields per group were measured.

### 2.10 RNA isolation and real‐time PCR analysis

Total RNA was extracted with the PureLink RNA Mini Kit according to the manufacturer's instructions (Ambion), followed by the cDNA synthesis using SuperScript VILO cDNA Synthesis Kit (Life technologies). Gene expression was measured by the 2^−ΔΔCT^ method using ABI7500 (Applied Biosystems) with the ABI TaqMan primer sets as specified in the supporting information.

## 3 RESULTS

### 3.1 Development of the LOXL2/LOXL3 inhibitor PXS‐5153A

PXS‐5153A (Figure S2) is an innovative dual inhibitor of LOXL2/LOXL3, the design of which was based on biochemistry of the target enzyme(s) coupled with previously gained knowledge of fluoroallylamine‐bearing, mechanism‐based inhibitors.^29^

PXS‐5153A exhibited an IC_50_ of <40 nmol/L for LOXL2 across all mammalian species tested (Table 1). PXS‐5153A also inhibited human LOXL3 with an IC_50_ value of 63 nmol/L. The compound is >40‐fold selective for LOXL2 over both LOX and LOXL1 and >700‐fold selective over other related amine oxidases. PXS‐5153A was found to have little to no activity against a number of additional targets (Table S1), with the exception of Adrenergic α2A (97%, when tested at 10 μmol/L) and calcium channel l‐type, dihydropyridine receptor, rat (80%, when tested at 10 μmol/L).

**Table 1:** Activity and selectivity of PXS‐5153A. Each value is an average of at least three experiments

Assay	pIC_50_ ± SD (IC_50_ nmol/L)
Recombinant human LOXL2	7.7 ± 0.23 (21)
Recombinant mouse LOXL2	7.7 ± 0.2 (21)
Recombinant rat LOXL2	7.8 ± 0 (15)
Recombinant dog LOXL2	8.04 ± 0.05 (9)
Native human LOXL2	7.4 ± 0.12 (38)
Native human LOX	5.7 ± 0.16 (1790)
Recombinant human LOXL1	5.8 ± 0.23 (1408)
Recombinant human LOXL3	7.2 ± 0.23 (63)
Recombinant human LOXL4	7 ± 0.1 (104)
SSAO, MAO‐A and MAO‐B	(>30 000)

PXS‐5153A was designed to interact with the LTQ (lysine tyrosylquinone) cofactor in the enzymatic pocket of LOXL2 and LOXL3, which, upon elimination of the fluoride‐leaving group, leads to a covalently bound enzyme‐inhibitor complex. The importance of the leaving group was highlighted by the 20‐fold reduced potency displayed by the corresponding des‐fluoro analogue (Table S2). PXS‐5153A is a mechanism‐based inhibitor that irreversibly blocks enzymatic function with an apparent binding constant (Ki) of 1.01 μmol/L and rate of inactivation (kinact) of 0.20/minute.

**Figure 1:**
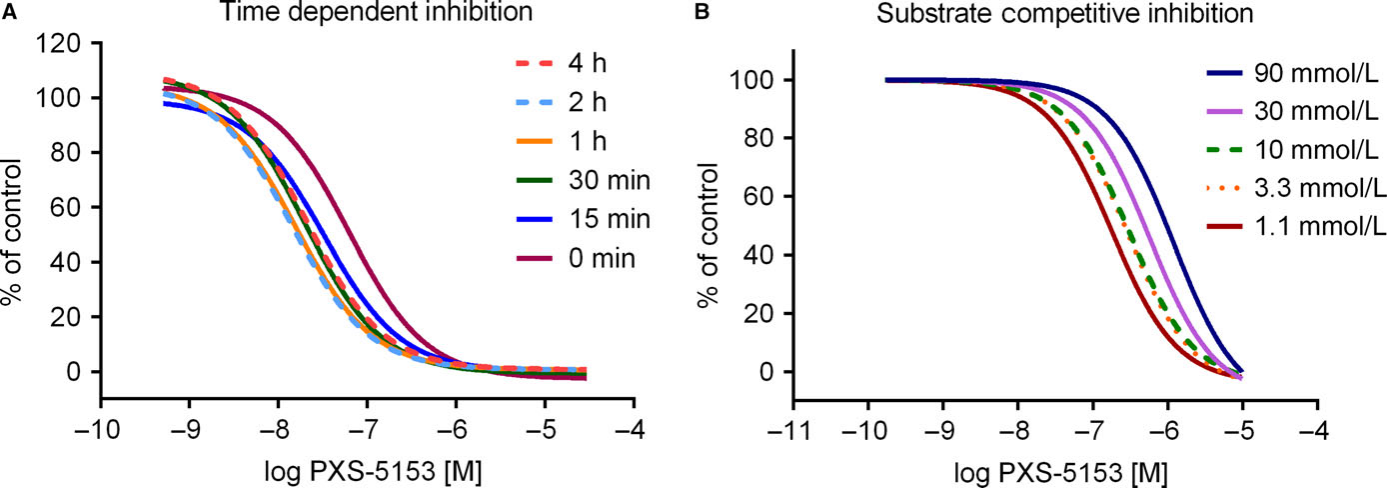
Mechanism‐based inhibition of PXS‐5153A. (A) Concentration and time dependent inhibition of LOXL2 activity by PXS‐5153A, wherein PXS‐5153A displays increased potency upon longer incubation with the enzyme. (B) Substrate concentration‐dependently diminished the potency of PXS‐5153A consistent with substrate competition for the LOXL2 enzymatic site

Further experimental evidence in support of the mechanism of action includes (a) time‐dependent LOXL2 inhibition, wherein PXS‐5153A displayed increased potency upon longer incubation with the enzyme (Figure 1A); (b) substrate competition, in which increasing concentrations of the substrate reduced inhibition by PXS‐5153A in a competitive manner (Figure 1B) and (c) jump dilution experiments, in which a 100‐fold dilution from 30 × IC_50_ of the inhibitor led only to a small recovery (30%) in enzyme activity of PXS‐5153A and almost full recovery of des‐fluoro analogue of PXS‐5153A (Figure S3A). As PXS‐5153A inhibits LOXL2 in a two‐step process by first binding to the LTQ and subsequent formation of an additional bond, the small recovery is likely to be related from PXS‐5153A not completely bound to the enzyme. Notably, when the jump dilution experiment was performed with LOXL1, enzymatic activity was fully recovered upon dilution (Figure S3A), confirming reversibility of inhibition of LOXL1.

PXS‐5153A is a fast acting inhibitor, with enzymatic activity almost entirely blocked within 15 minutes, as judged by the shift in the concentration response curves (Figure 1A). In contrast, the racemate of the selective LOXL2 inhibitor of PAT‐1251‐ requires approximately 4 hours to achieve complete inhibition (Figure S3B).

The pharmacokinetic (PK) properties of PXS‐5153A were investigated in rats and mice and are reported in Table S3. In rats, when dosed at 10/5 mg/kg (oral/i.v.), PXS‐5153A displayed a bioavailability of 10% and a half‐life of approximately 1.5 hours (i.v.). In mice, when dosed at 5 mg/kg (oral), PXS‐5153A displayed a bioavailability of 40% and a half‐life of approximately 1.1 hours (i.v.). Due to the high potency, it was reasoned that a single daily dose of 3‐10 mg/kg would be sufficient to achieve long‐lasting enzyme inhibition due to the fast acting, irreversible mechanism.

### 3.2 Inhibition of in vitro collagen oxidation and crosslinking by PXS‐5153A

**Figure 2:**
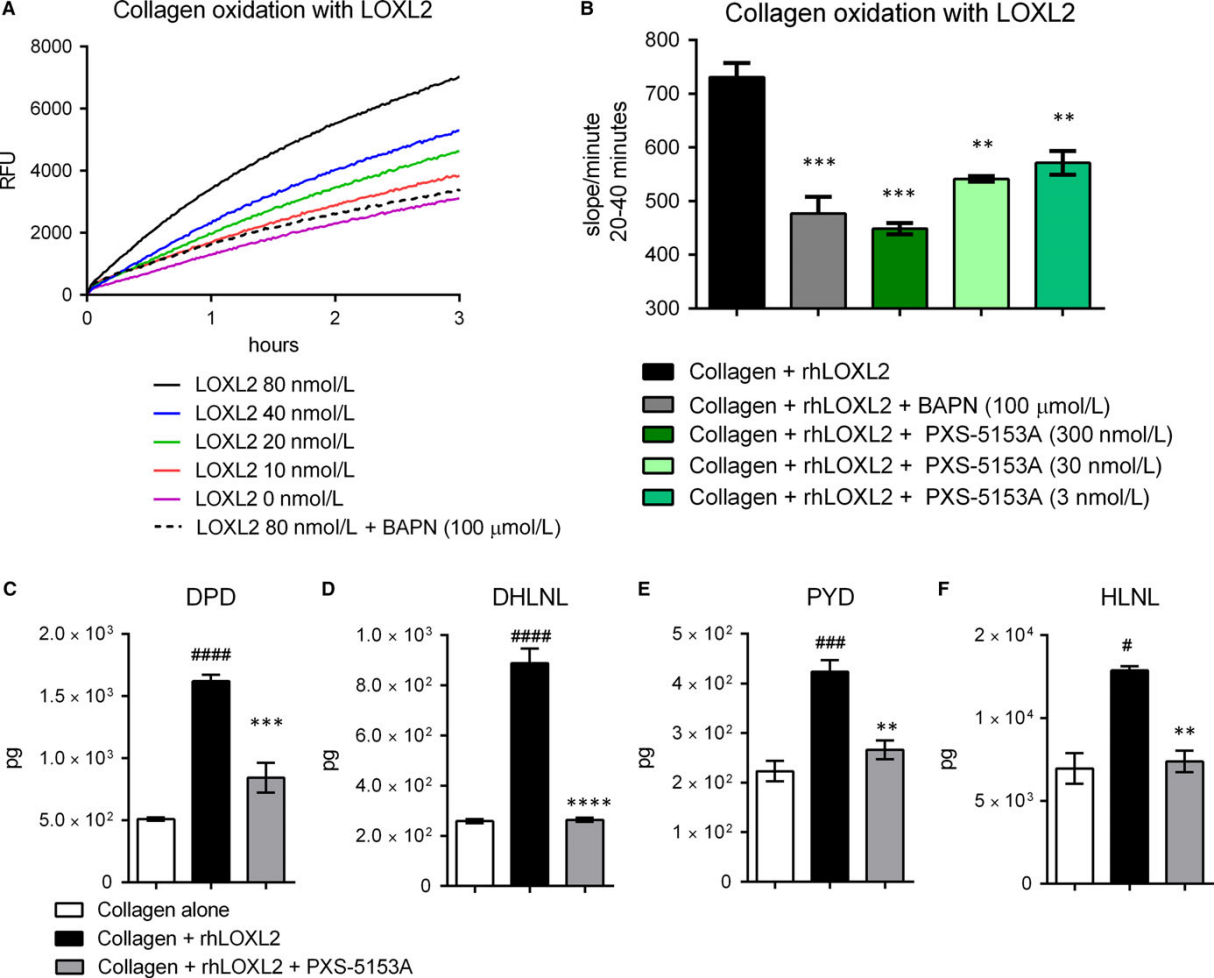
Collagen oxidation and crosslinking inhibition by PXS‐5153A. rhLOXL2 was incubated with a natural substrate (collagen) and oxidation was analysed using an AR/HRP assay with (A) 375 μg/mL of collagen used as a substrate. (B) Slope from the collagen oxidation assay using 20 nmol/L rhLOXL2 and different concentrations of PXS‐5153A at the 20‐40 minute time point. 20 nmol/L of rhLOXL2 was added daily to collagen for 5 days. 200 nmol/L of PXS‐5153A was pre‐incubated with rhLOXL2 for 30 minutes before addition to collagen. Data are presented as means ± standard deviation and differences between groups were assessed using one‐way ANOVA followed by Dunnett's test. Total crosslinks were analysed by LCMS/MS on day 7. (C) DHLNL. (D) Pyridinoline. (E) HLNL. (F) Deoxypyridinoline. Data are presented as mean values ± SEM. Data are compared using Student Two‐tailed *t* test. **P < 0.01, ***P < 0.001, ****P < 0.0001 compared to collagen + rhLOXL2; ^#^ *P* < 0.05, ^###^ *P* < 0.001, ^####^ *P* < 0.0001 compared to collagen alone

With the successful development of the dual LOXL2/LOXL3 enzymatic inhibitor PXS‐5153A, it was feasible to begin exploring the role of these enzymes during the collagen crosslinking process. To confirm the positive effect of the small molecule inhibitor in the initial steps of this process, collagen oxidation was assessed after incubation with enzymatically active rhLOXL2. As expected, rhLOXL2 dose dependently induced oxidation of collagen (Figure 2A) with PXS‐5153A dose‐dependently impeding collagen oxidation (Figure 2B).

Given the ability of PXS‐5153A to hinder the initial step of crosslinking, it was anticipated that crosslink formation would also be ablated. To confirm this hypothesis, purified collagen was incubated with rhLOXL2 enzyme for 7 days and crosslinks were measured by LCMS/MS. As was anticipated, rhLOXL2 increased the formation of crosslinks (Figure 2C‐F) with significant increases in the immature crosslinks DHLNL and HLNL as well as the mature crosslinks PYD and DPD. Notably, treatment with PXS‐5153A prevented all crosslink formation.

### 3.3 Inhibition of CCl_4_‐induced fibrosis by PXS‐5153A

**Figure 3:**
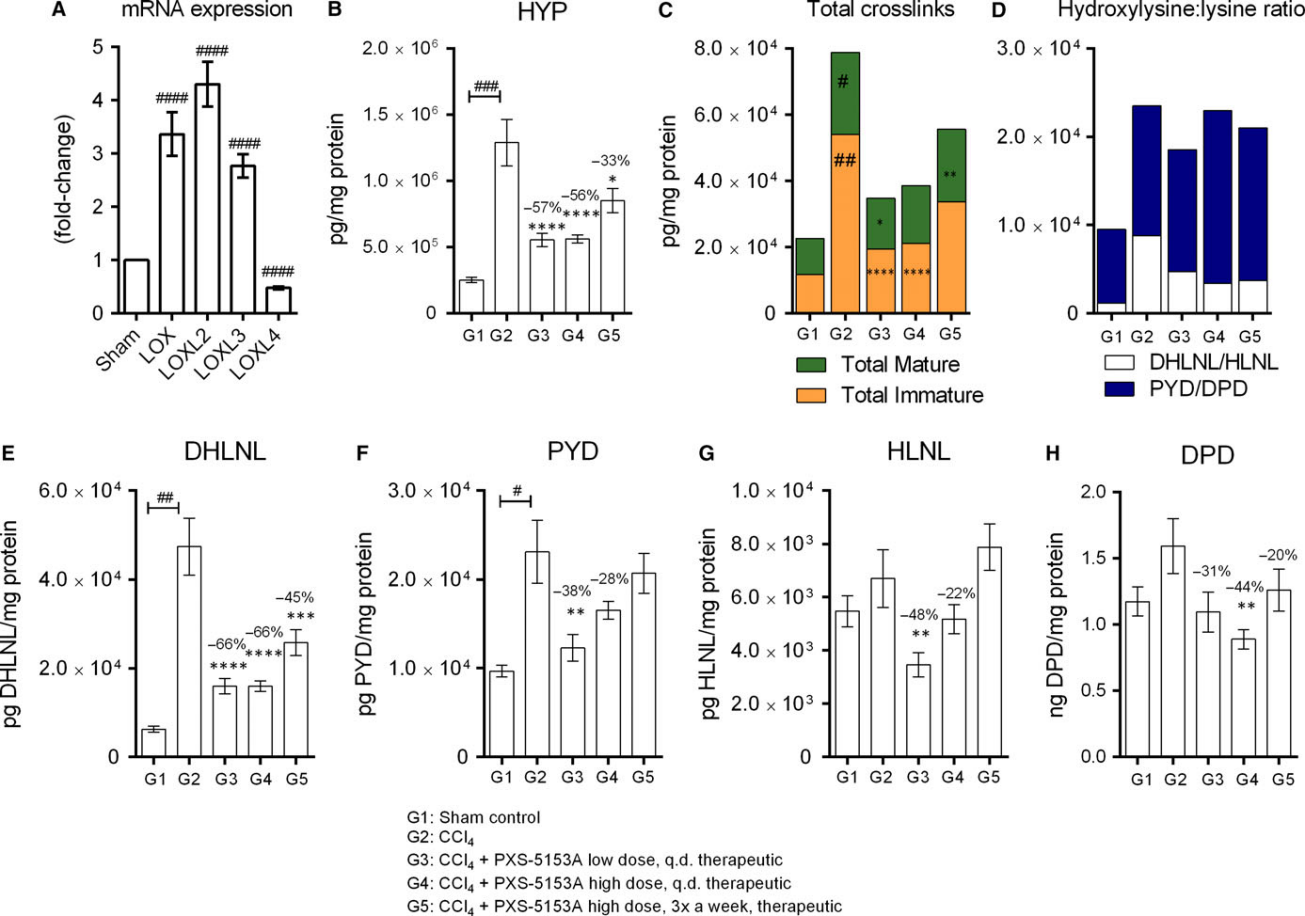
(A) The mRNA expression of LOX family members was assessed after 6 weeks of CCl_4_ treatment. (B‐H) In vivo crosslink inhibition by PXS‐5153A. Animals underwent a CCl_4_ liver fibrosis model and crosslinks were quantified in the livers by LCMS/MS (B) inhibition of total hydroxyproline by PXS‐5153A. (C) Inhibition of total immature collagen crosslink density (DHLNL + HLNL) as well as total mature crosslink density (PYD + DPD) by PXS‐5153A. (D) Hydroxylysine:lysine ratio. (E) DHLNL. (F) Pyridinoline. (G) HLNL. (H) Deoxypyridinoline. Data are presented as mean values ± SEM for n = 6 Sham and n = 14‐15 CCl_4_. Data are compared using Student Two‐tailed *t* test. *P < 0.05, **P < 0.01, ***P < 0.001, ****P < 0.0001 compared to the CCl_4_ group; ^#^ *P* < 0.05, ^##^ *P* < 0.01, ^###^ *P* < 0.001, ^####^ *P* < 0.0001 compared to the sham control

To confirm that the PXS‐5153A‐mediated inhibition of crosslinking seen in the in vitro assay would ensue in an in vivo setting, the CCl_4_ model of liver fibrosis—that can be LOXL2 dependent—was performed.^13^ Remarkably, mRNA levels of LOXL2 and LOXL3 were substantially increased upon 6‐weeks of CCl_4_ treatment (Figure 3A), which confirmed the suitability of the model for assessing the role of LOXL2/LOXL3.

To verify the induction of fibrosis in these animals, hydroxyproline (HYP) a marker of collagen content was analysed. The CCl_4_‐treated control group showed a significant increase in HYP as compared to healthy controls (Figure 3B); while all dosing regimens of PXS‐5153A significantly reduced the HYP content by >30%.

Total immature collagen crosslink as well as total mature crosslink quantities were then analysed. There was a significant increase of immature as well as mature crosslinks upon CCl_4_‐stimulus compared to healthy animals (4.6 and 2.7‐fold increase, respectively; Figure 3C). Therapeutic treatment of PXS‐5153A substantially reduced immature crosslink formation compared with the CCl_4_ treated animals. Mature crosslink formation was also reduced by PXS‐5153A treatment, however due to variability of the signal only low dose reached significance.

The contribution and effect on each individual type of crosslink was next assessed. The formation of the immature crosslink DHLNL showed dominance over all the other crosslinks, with a 7.6‐fold increase in the CCl_4_‐treated control group compared to 2.4‐fold of PYD and only slight increases of DPD and HLNL (Figure 3E‐H). All groups with therapeutic treatment of PXS‐5153A showed a significant reduction in DHLNL formation compared to the CCl_4_ treated animals. Similarly, all groups with PXS‐5153A treatment showed different degrees of reduction in PYD, however only high dose q.d. was statistically significant. Even though there were only slight increases of DPD and HLNL by CCl_4_, high dose q.d. treatment of PXS‐5153A significantly reduced DPD crosslinks.

The pathway dominance (hydroxyallysine or allysine‐derived crosslinks) can be determined by calculating the ratio between hydroxylysine:lysine crosslinks and is a potential indicator of fibrotic status. The ratio was elevated in all CCl_4_‐treated animals due to increased levels of DHLNL and PYD (Figure 3D).

To determine the rate of crosslink formation compared to the secretion of collagen, the quantities of each crosslink was normalized relative to the hydroxyproline content. Upon CCl_4_ treatment, the ratio between DHLNL/HYP was increased, indicating that the formation of DHLNL is faster than the synthesis and secretion of collagen, and this effect was reversed by treatment with PXS‐5153A (Figure S4A). In contrast, the ratios between PYD/HYP, HLNL/HYP and DPD/HYP all decreased, demonstrating the slow rate of formation of these crosslinks compared to the fast secretion of collagen (Figure S4B‐D).

**Figure 4:**
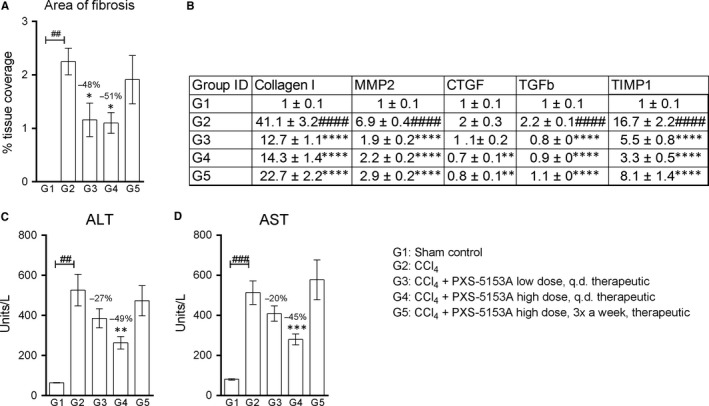
Decreased CCl_4_‐induced liver fibrosis by PXS‐5153A. (A) Quantitative analysis of area of fibrosis by collagen staining with Picrosirius red. (B) mRNA expression of fibrotic markers in whole liver homogenates using Taqman gene expression assays, data normalised to GAPDH and presented as fold change over sham control. Quantities of (C) Alanine aminotransferase and (D) aspartate aminotransferase in experimental liver fibrosis was determined in blood. Data are presented as mean values ± SEM for n = 6 Sham and n = 14‐15 CCl_4_. Data are compared using Student Two‐tailed *t* test. *P < 0.05, **P < 0.01, ***P < 0.001, ****P < 0.001 compared to the CCl_4_ group; ^##^ *P* <  0.01, ^###^ *P* < 0.001,^ ####^ *P* <  0.001 compared to the sham control group

Tissue collagen quantification by Picrosirius red staining represents another important tool in the diagnosis of liver injury/fibrosis as it allows qualitative and quantitative evaluation of collagen fibers.^30^ In the CCl_4_‐treated animals, the amount of fibrillar collagen was markedly augmented by disease, as reflected in the 2.4‐fold increase in percentage coverage area by Picrosirius red staining (Figure 4A). PXS‐5153A strongly reduced collagen accumulation by up to 51% in both of the daily dosing groups. Furthermore, PXS‐5153A treatment suppressed the induction of a network of key fibrotic marker genes in the liver (Figure 4B).

To understand whether inhibition of LOXL2/LOXL3 enzymatic functions would translate to liver function improvements, disease severity was examined upon treatment with PXS‐5153A. ALT and AST are hepatic enzymes that are excessively released into the bloodstream upon liver damage, and provide an indirect measurement of liver function and hepatocyte impairment.^31^ Upon CCl_4_ treatment, liver injury was severe and significant increases in ALT and AST were detected, when compared to healthy animals (8.2 and 6.3‐fold increase, respectively; Figure 4C/D). Liver injury was significantly dampened by PXS‐5153A high dose q.d. treatment compared with the CCl_4_ treated animals.

### 3.4 Inhibition of NASH by PXS‐5153A

Although the CCl_4_ model recapitulates some of the histological features of liver fibrosis, it was important to determine whether LOXL2/LOXL3 enzymatic functions were also important for crosslink formation in a more physiologically relevant model. As such, animals underwent the STAM induced NASH model (recognized as exhibiting physiological fidelity to the human condition)^32^ and animals were then treated with PXS‐5153A.

**Figure 5:**
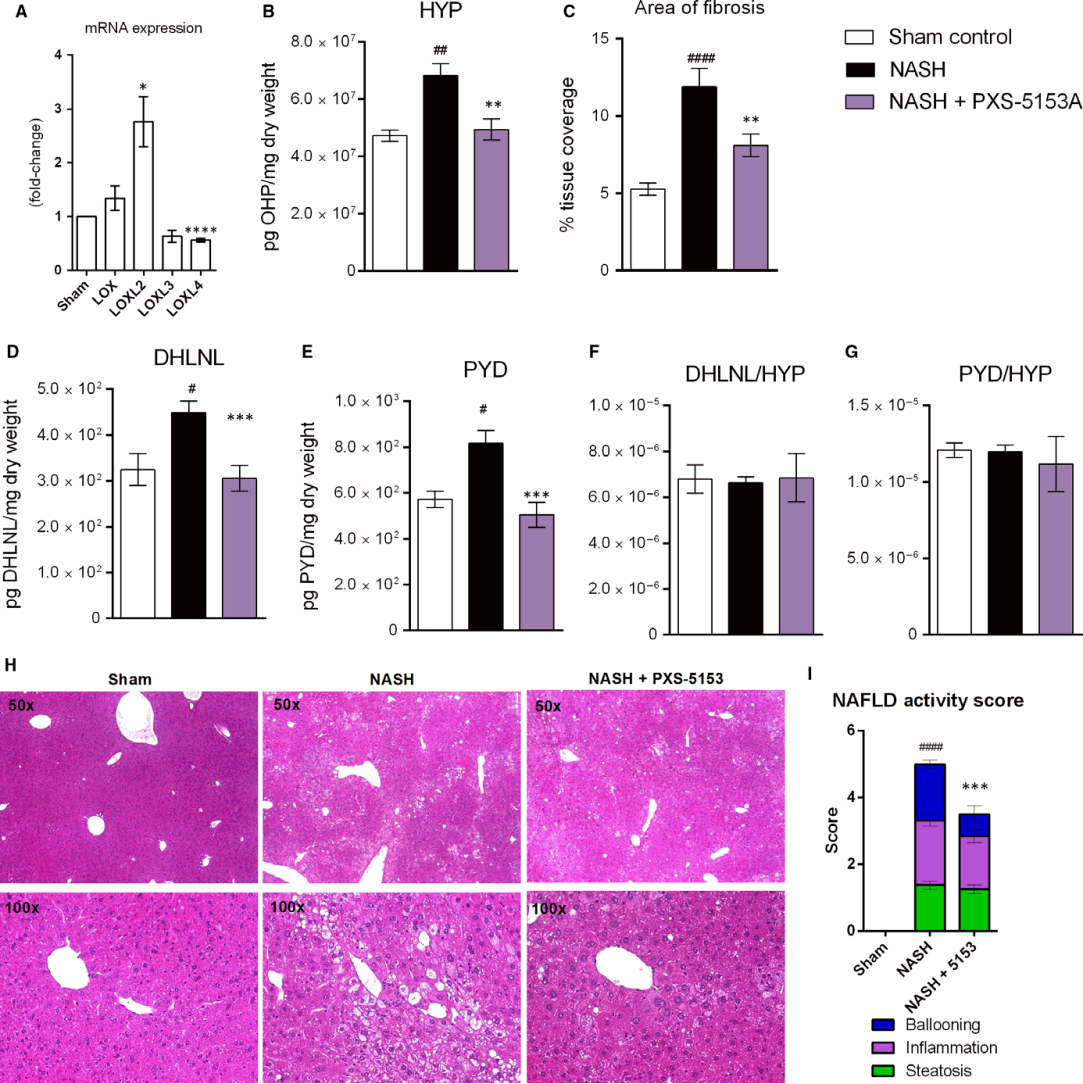
Inhibition of fibrosis by PXS‐5153A during the NASH model. (A) The mRNA expression of LOX family members was assessed after NASH induction. Collagen content was analysed in livers (B) by hydroxyproline analysis by LCMS/MS. (C) Area of fibrosis was analysed by staining with Picrosirius red. Mature and immature collagen crosslinks were quantified using LCMS/MS (D) DHLNL and (E) pyridinoline (PYD) and ratios over hydroxyproline (F) DHLNL/Hydroxyproline and (G) PYD/Hydroxyproline. Data are presented as mean values ± SEM for n = 6 Sham and n = 12‐16 NASH. Data are compared using Student Two‐tailed *t* test. *P < 0.05, **P < 0.01, ***P < 0.001 relative to the NASH group; ^#^ *P* < 0.05, ^##^ *P* < 0.01, ^####^ *P* < 0.01 compared with sham control. Effect of PXS‐5153A on NASH: (H) representative photomicrographs of the HE stained liver sections (50 and 100X); (I) NAFLD activity score (NAS), calculated according to the criteria of Kleiner (30). Data are presented as mean values ± SEM for n = 6 Sham and n = 12‐16 NASH. Histological scoring analysed using nonparametric Mann‐Whitney *U* test. Data are compared using Student Two‐tailed *t* test. ***P < 0.001, ****P < 0.001 relative to NASH. ^####^ *P* < 0.0001 compared with sham control

The gene expression profile of NASH livers showed a substantial increase in LOXL2 when compared to sham control animals (Figure 5A). Hydroxyproline (HYP) content was then analysed to verify the induction of fibrosis. Upon NASH induction, there was a 1.5‐fold increase in HYP compared healthy controls (Figure 5B). Treatment with PXS‐5153A caused a significantly reduction in HYP compared to the CCl_4_ group. In addition, the amount of fibrillar collagen was markedly augmented by disease as seen by the 2.2‐fold increase in percentage coverage area by Picrosirius red staining, which was reduced by PXS‐5153A (Figure 5C).

The contribution of LOXL2/LOXL3 enzymatic functions on individual crosslinks was then assessed. DHLNL and PYD were increased in the NASH animals by 1.5 and 1.9‐fold (Figure 5D/E), while treatment with PXS‐5153A significantly reduced these crosslinks. The ratio between each crosslink and HYP was also measured and found to be similar between NASH animals and healthy animals, indicating that both DHLNL and PYD formation paralleled collagen secretion (Figure 5F/G).

Histological analysis was then performed to understand the morphological changes induced by NASH and whether inhibition of crosslinking would affect these changes. Livers from the NASH group exhibited increased fat deposition, hepatocellular ballooning and inflammatory cell infiltration compared with the sham control group (Figure 5H). PXS‐5153A treatment significantly reduced hepatocyte ballooning demonstrating the hepatoprotective effects of the compound and was paralleled by a reduction in NASH disease score (Figure 5I).

### 3.5 Correlation between collagen crosslinks and liver function

To investigate whether there was a correlation between collagen crosslink amounts and liver function, a simple linear regression model was applied to directly compare the individual crosslinks with various liver functional readouts.

In the CCl_4_ model, there was a significant positive correlation between mature crosslinks and all liver functional readouts, while for the immature crosslinks there was only significant correlation with the percentage fibrotic (Figure S5A‐C). The contribution of PYD outweighed that of DPD and the contribution of DHLNL prevailed over that of HLNL (Figure S6A‐L).

In the NASH model, both PYD and DHLNL showed a significant positive correlation compared to the percentage fibrotic, and a modest correlation with the NASH score (Figure S7A‐D).

Overall, these results indicate that PYD and DHLNL can be used as predictors of disease severity during liver fibrosis, with levels of PYD showing more significance than those of DHLNL.

### 3.6 Inhibition of heart fibrosis by PXS‐5153A

**Figure 6:**
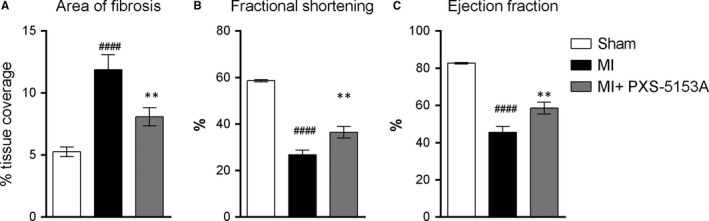
PXS‐5153 ameliorated myocardial infarction. (A) Quantitative analysis of area of fibrosis by collagen staining with Picrosirius red. (B) Fractional shortening. (C) Ejection fraction. Data are presented as mean values ± SEM for n = 11 Sham and n = 14‐16 MI. Data are compared using Student Two tailed *t* test. **P < 0.01 relative to MI group. ^####^ *P* < 0.001 compared to the sham control

A number of studies have indicated that inhibition of LOX family members, in particular LOXL2, can positively influence myocardial remodelling,^33^, ^34^ with LOXL2 mRNA expression being highly upregulated upon cardiac disease.^34^ As such, the impact of LOXL2/LOXL3 enzymatic inhibition was studied in a model of post‐myocardial infarction remodelling. Several functional readouts demonstrated that inhibition of LOXL2/LOXL3 by PXS‐5153A resulted in a remarkable interference in disease progression, with a decrease in percentage fibrotic coverage area, improvement in fractional shortening as well as ejection fraction compared to infarcted untreated animals observed (Figure 6A‐C).

## 4 DISCUSSION

Lysyl oxidases have been proposed to play a crucial role in the development of fibrosis owing to their ability to crosslink collagen within the fibrotic matrix.^2^ A novel selective LOXL2/LOXL3 dual inhibitor, PXS‐5153A, was developed to evaluate the specific role of the enzymatic function of these two enzymes in collagen crosslinking and fibrosis. In line with previous reports, LOXL2 was found to play a significant role in fibrosis,^13^, ^14^ with mRNA upregulation during liver fibrosis. Furthermore, these findings extended to LOXL3, which previously had only been linked to lung fibrosis.^2^

PXS‐5153A is an irreversible inhibitor, which, upon elimination of the leaving group, leads to a covalently bound enzyme‐inhibitor complex. It is fast‐acting, affording complete inhibition of the enzyme within 15 minutes at 10xIC_50_. Importantly it fulfils all criteria for mechanism‐based inhibition of LOXL2 and LOXL3, as it displays time‐dependent inhibition, substrate competition and only a small recovery in activity after jump dilution experiments. Notably, enzymatic activity was fully restored when the recovery experiment was performed with LOXL1 (used as a surrogate for LOX owing to similar pharmacology), thereby increasing confidence in the potential safety window of the compound. Using a natural substrate (collagen) in an oxidation assay as well as a crosslinking assay, PXS‐5153A demonstrated the expected behaviour and potency.

Upon LOXL2/LOXL3 inhibition, liver fibrosis was reduced during CCl_4_ stimulus and STZ‐high fat diet induced NASH. PXS‐5153A reduced disease severity and improved organ function by diminishing collagen content, excessive collagen crosslinking and maturation. PXS‐5153A diminished, but did not abolish, healthy collagen deposition nor did it ablate immature crosslink formation, potentially owing to the fact that LOX and LOXL1 are not irreversibly inhibited. Consequently, PXS‐5153A potentially offers a beneficial approach to the treatment of fibrosis as it allows healthy tissue repair, thereby leading to gradual and persistent degradation of fibrillar scar without having a significant impact on collagens that are part of the normal tissue architecture. It is noteworthy that although the association of lysyl oxidase functional enzymatic activity in crosslinking have been widely proposed, this is the first study that corroborates that complete enzymatic inhibition of LOXL2 and LOXL3 diminishes fibrosis in a variety of models due to the aforementioned mechanism. It is important to highlight that PXS‐5153A also showed a remarkable hindrance in cardiac disease progression with inhibition of mechanical dysfunction of infarcted hearts.

On a more fundamental level, the findings of this study confirm that hydroxyallysine‐derived crosslinks are a direct result of LOXL2/LOXL3 enzymatic function. The mechanism by which collagen crosslinks are formed is based on the reactions of allysine or hydroxyallysines present on collagen side‐chains with other aldehydes or with unmodified lysine or hydroxylysine residues, resulting in the formation of crosslinks.^7^, ^9^, ^10^, ^11^ The availability of hydroxyallysine is the direct result of lysine hydroxylation, through a process driven by lysyl hydroxylases.^35^ In bone, tendon, ligaments and cartilage,^36^, ^37^ the collagen is crosslinked mainly via the hydroxyallysine route, whereas in skin the crosslinks are derived from the allysine route.^38^, ^39^ During disease however, there are changes in crosslink pathway preferences as well as crosslinks quantities. The pattern of crosslinks in alcoholic cirrhosis and alveolar echinococcosis^40^ as well as viral cirrhotic liver and chronic viral hepatitis^41^ are characterized by an increase in pyridinolines (hydroxyallysine‐derived). With a similar pattern being reported in irreversible localized scleroderma,^42^ irreversible keloids^43^ and pulmonary fibrosis.^44^, ^45^ Interestingly, in hypertrophic scars, during the initial stages of wound healing there is a strong preference towards allysine crosslinks, which changes over time to that of the hydroxyallysine crosslinks.^38^ Taken together, the findings consistently substantiate that hydroxyallysine crosslinking is the principal pathway involved during fibrosis. These findings are also in agreement with the current study, as it showed the preferential contributions of DHLNL/PYD in CCl_4_ induced liver fibrosis as well as NASH induced STZ‐high fat diet. Given that overhydroxylation of lysine is a consequence of increases in the levels of lysyl hydroxylase,^42^, ^46^, ^47^, ^48^ it is conceivable that availability of lysyl hydroxylases dictates the pattern of crosslinking during disease.

Even though crosslink maturity as well as allysine/hydroxyallysine pathway ratios have been previously correlated with tissue mechanical properties,^49^, ^50^ this is the first study to correlate hydroxyallysine pathway with liver organ function during fibrosis. ALT and AST are commonly used as predictors of liver function, as the release of these enzymes from liver cells to the bloodstream parallels hepatocellular damage or death and provide an indirect measurement of liver and hepatocyte impairment.^31^ In the CCl_4_ model, there was a positive correlation between mature crosslinks and all functional readouts (ALT, AST and percentage fibrotic coverage area), clearly showing that LOXL2/LOXL3‐mediated crosslinks diminish organ function during fibrosis.

A number of studies have been useful in confirming the role of lysyl oxidases in controlling tissue stiffness and collagen extractability,^14^, ^51^, ^52^ however, they did not provide definite evidence for the correlation between lysyl oxidase enzymatic action and collagen crosslinking. In our current study, analysis of the liver revealed that the content of immature (DHLNL and HLNL) and mature (PYD and DPD) crosslinked collagen was increased upon the induction of liver fibrosis and that dual inhibition of the enzymatic functions of both LOXL2/LOXL3 reduced formation of these crosslinks. Although a direct comparison of individual crosslinks and liver stiffness was not performed in the current study, the results generated support the concept that difficulty in extractability and increases in tissue stiffness^14^, ^52^ are due to collagen crosslink maturation. Altogether, these results suggest that LOXL2/LOXL3 enzyme‐dependent crosslink formation leads to remodelling during fibrosis and, overall, tougher tissue.

Taken together, these results show that LOXL2 and LOXL3 enzymatic functions are key players in the formation of hydroxyallysine derived collagen crosslinks during fibrosis. This study highlights the potential of inhibiting LOXL2/LOXL3 enzymatic activities, for example by use of PXS‐5153A, as a novel therapeutic tool for the treatment of diseases that are characterized by abnormal increases in collagen crosslinking.

## AUTHORS CONTRIBUTION

H.S., A.D.F. and W.J. involved in conception and design. H.S., A.D.F., L.P., T.T.Y., J.M., A.Z., C.I.T., M.D., J.S.F., W.Z., A.G., A.J., B.J., S.T. and A.B. involved in acquisition and analysis. H.S., A.D.F., W.J., A.B., A.G. and L.P. involved in interpretation. All authors approved the final manuscript.

## CONFLICT OF INTEREST

The project was fully funded by Pharmaxis. During the study HS, ADF, LP, TTY, JM, AZ, CIT, MD, JSF, WZ, AG, AJ, AB and WJ were Pharmaxis employees and share‐holders. Compound PXS‐5153A tested in the current study is owned by Pharmaxis.
