# Supplementary material for: The lysyl oxidase like 2/3 enzymatic inhibitor, PXS‐5153A, reduces crosslinks and ameliorates fibrosis
Source: J Cell Mol Med. 2018 Dec 9;23(3):1759–70. doi: 10.1111/jcmm.14074 (PMC6378217; doi:10.1111/jcmm.14074)
Supplement: Supplementary file 1 [file JCMM-23-1759-s001.docx]

**The lysyl oxidase like 2/3 enzymatic inhibitor, PXS-5153A, reduces crosslinks and ameliorates fibrosis**

**Supporting information: Materials and methods**

Fluorometric Enzymatic Activity Assays

The measurement of the enzymatic activity of all lysyl oxidase family members was based on the detection of hydrogen peroxide with an Amplex-Red oxidation assay, as described in Zhou et al.[[1](#_ENREF_1)]; miniaturised in 384 well format, with the appropriate combination of substrate and assay buffer for each individual enzyme. After a 30 minute pre-incubation of the enzyme at 37 °C with the test compound (or with different incubation times for the time-dependency assay), one volume of reaction mixture containing 120 μM Amplex Red (AR) (Life Technologies), 1.5 U/mL horseradish peroxidase (HRP) (Sigma-Aldrich) and the specific substrate was added to each sample. The relative fluorescence units (RFU) were then measured every 2.5 minutes for 30 minutes at 37°C, excitation 565 nm and emission 590, on a BMG Clariostar Microplate Reader. All the substrates were used at concentrations corresponding to their Km towards the corresponding enzyme, or with a range of different concentrations for the substrate competition experiments.

Lysyl oxidases assay buffer contained 1.2 M urea, 50 mM sodium borate buffer, pH 8.2 and 100 µM β-aminoproprionitrile (BAPN, Sigma-Aldrich) was used for low control.

Recombinant human LOXL1 was expressed and purified from cDNA (GeneArt) and 10 mM putrescine was used as substrate. Recombinant human and mouse LOXL2 (R&D Systems) were challenged with 5 mM putrescine (Sigma-Aldrich) as the substrate. Rat and dog recombinant LOXL2 were amplified and purified in house from HEK-293 clones kindly provided by Dr. Fernando Rodríguez Pascual, Madrid University, and used in the same conditions as for the human form. 2 mM putrescine was used as the substrate for recombinant human LOXL3 (R&D Systems) and LOXL4 (Dr. Fernando Rodríguez Pascual, university of Madrid, Spain) assays.

Native human LOX and LOXL2 were obtained from the conditioned medium of IMR90 cultures (ATCC), after 3 days in DMEM (0.1% FBS, no phenol red, supplemented with non-essential amino acids and 10 µM CuSO_4_). The supernatant was concentrated and buffer exchanged to 4 M urea, 50 mM sodium borate buffer, pH 8.2 by means of Amicon 10-kDa centrifuge filters (Millipore). The concentrated supernatant was then fractionated with Amicon filters with a higher cut-off (50-kDa), separating LOXL2 in the portion at higher molecular weight, and collecting the 30-kDa LOX in the lower molecular weight portion. The two enzymes were further concentrated according to the assay requirements, buffer exchanged with Amicon filters to 1.2 M urea, 50 mM sodium borate buffer, pH 8.2. The enzymes were subsequently verified by means of Western Blot and against a set of selective inhibitors with known pharmacology. 10 mM or 5 mM putrescine were used as substrates for native LOX and native LOXL2, respectively.

Recombinant human semicarbazide sensitive amine oxidase (SSAO/VAP1), diamine oxidase (DAO) and monoamine oxidases A and B (MAO-A and MAO-B) assays were performed as previously described [[2](#_ENREF_2)].

*Jump dilution assay*

The measurement of the residence time was based on the detection of hydrogen peroxide with an Amplex-Red oxidation assay, as described in Copeland et al. [[3](#_ENREF_3)] in a 96 well format. The target was incubated with the test compound at 30 x IC50 for 40 minutes at 37 °C in snapstrip PCR vials. After the incubation, a 100-fold dilution is carried out in lysyl oxidase assay buffer into the vials. The diluted enzyme-inhibitor complex is added to a 96 well plate followed by the addition of the reaction mixture (20 mM putrescine for LOXL1, 10 mM putrescine for LOXL2). The target percentage activity is measured as a function of time after the dilution event.

*Time dependency*

Time-dependent inhibition of the target by the compound was assessed by pre-incubation of 25 uL of target enzyme at 37 °C in a 384-well plate. An 11-point dilution was carried out of the test compound followed by its addition at 0.5 uL to the enzyme plate at different time points (0 minutes, 15 minutes, 30 minutes, 1hour, 2 hours and 4 hours unless otherwise stated in the graph). At the last time point, the reaction mixture was added and the relative fluorescence was measured.

*Substrate competition*

The measurement of substrate competition was based on the release of hydrogen peroxide as previously described. The target enzyme was incubated with the inhibitor, and differing concentrations of the substrate (putrescine) in reaction mixture and measured.

Collagen oxidation assay

Collagen oxidation assay was based on the release of hydrogen peroxide as described previously described [[2](#_ENREF_2)]. In a 384 black well plate, 25 μL of collagen (rat tail, type I, Thermo Fisher, 1.5 mg/mL in 50 mM sodium borate buffer, pH 8.2) was combined with 25 μL rhLOXL2 (R&D Systems) with or without the pan-lysyl oxidase inhibitor BAPN (100 μM, Sigma-Aldrich) or PXS-5153A. A reaction mixture using AR (120 μM; Life Technologies), HRP (1.5 U/mL; Sigma-Aldrich) was prepared in 50 mM sodium borate buffer. 50 μL of the reaction mixture was added into each well. The relative fluorescence units (RFU) were read every 1 minute for 3 hours at 37°C, excitation 565 nm and emission 590 (Clariostar, BMG labtech). The slope per minute of the kinetic curves for each sample was calculated using MARS data analysis software (BMG labtech) in the linear phase (between the 20-40 minute time points).

Protein, hydroxyproline and collagen crosslinking analysis

Samples were freeze-dried and homogenized until a powdered sample was obtained. 10 mg of sample was placed in acid and heat resistant vials and reduced with NaBH_4_ (0.4 mg/mL in 0.6 mM NaOH) for 30 minutes at room temperature, reaction was stopped by adding 50 µL neat acetic acid. Sample was pelleted by centrifugation and washed three times with MiliQ H_2_O. Pellet then underwent acid hydrolysis in 6 M HCl at 100 ^o^C for 24 hours. Hydrolyzed samples were evaporated to dryness under vacuum and then resuspended in HPLC-grade water (protein content was assessed at this stage). Crosslinks were extracted from the hydrolysate using an automated solid phase extraction system (Gilson GX-271 ASPECA system) employing reversed-phase C18-Aq columns (GracePure, Thermo Fisher) followed by strong cation exchange columns. After extraction and drying, the crosslinks were converted into heptafluorobutyric acid (HFBA) salts for analysis by UHPLC-ESI-MS/MS on a Thermo Dionex UHPLC and TSQ Endura triple quad mass spectrometer. UHPLC separation of crosslinks was achieved with an Agilent Rapid Resolution High Definition SB-C18 column. UHPLC was performed using a 12 minute gradient flow of the mobile phase A (10 mM ammonium formate, 0.1% formic acid, 0.1% HFBA in H_2_O) from 96.2 to 0% and mobile phase B (10 mM ammonium formate, 0.1% formic acid, 0.1% HFBA in 80% MeOH) from 3.8 to 100% at a flow rate of 0.3 mL/min and with a column temperature of 40 °C. Positive ESI-MS/MS with Selected Reaction Monitoring mode was performed using the following parameters: Spray voltage 4000 V; sheath gas 35 (Arb); aux gas 20 (Arb); sweep gas 0 (Arb); ion transfer tube temperature 350 °C; vaporizer temperature 300 °C. Standards used for quantitation of collagen crosslinks or total collagen in hydrolysates by UHPLC-ESI-MS/MS were DHLNL (Thermo Fisher), HLNL (Toronto Research Chemicals, Inc), PYD and DPD (Quidel Corporation) and hydroxyproline (Sigma-Aldrich). Quantitation of the collagen crosslinks and total hydroxyproline was achieved by comparing to a standard curve. Sample values were interpolated using GraphPad Prism 7 software. Total protein was quantified in the samples using a commercially available kit (QuickZyme Biosciences, Leiden, The Netherlands).

Data analysis on LCMS/MS

Tissue samples were analysed on LCMS/MS using three standard calibrations i.e. “bracketing calibration”. The basis of this strategy was to monitor the assay variability of detector response during the run. The variability for LCMS/MS detector response was observed by monitoring the % CV (Coefficient of Variance) of peak responses of adjacent calibrators injected before and after the tissue samples. When a clear trend of signal variability was observed before and after samples, the assay was considered as failed. The average calibration plot of the three standard calibrations was used for calculations of concentrations generated for each biomarker in an individual sample. An external standard method was used for quantitation due to the absence of internal standards in the method.

TaqMan primer sets

Data was normalised to expression of the internal quantitative control (GAPDH) and expressed as the fold-change compared to the Sham treatment group.

| **Gene Symbol** | **Gene** | **Assay Id.** |
| --- | --- | --- |
| lox | Lysyl oxidase | Rn01491829_m1 |
| loxl2 | Lysyl oxidase-like 2 | Rn01466080_m1 |
| loxl3 | Lysyl oxidase-like 3 | Rn01765241_m1 |
| loxl4 | Lysyl oxidase-like 4 | Rn01410872_m1 |
| col1a1 | Collagen, type I, alpha 1 | Rn01463848_m1 |
| mmp2  ctgf | Matrix metallopeptidase 2  Connective tissue growth factor | Rn01538170_m1  Rn01537279_g1 |
| tgfb1 | Transforming growth factor, beta 1 | Rn00572010_m1 |
| timp1  gapdh | Tissue inhibitor of metalloproteinase 1  Glyceraldehyde-3-phosphate dehydrogenase | Rn01430873_g1  Rn01775763_g1 |
|  |  |  |

**Supporting information: Figures**

Figure 1

Formation of intermediate reducible crosslinks (immature) and non-reducible crosslinks (mature) in collagen by reaction of allysine or hydroxyallysine in the telopepetide domain.


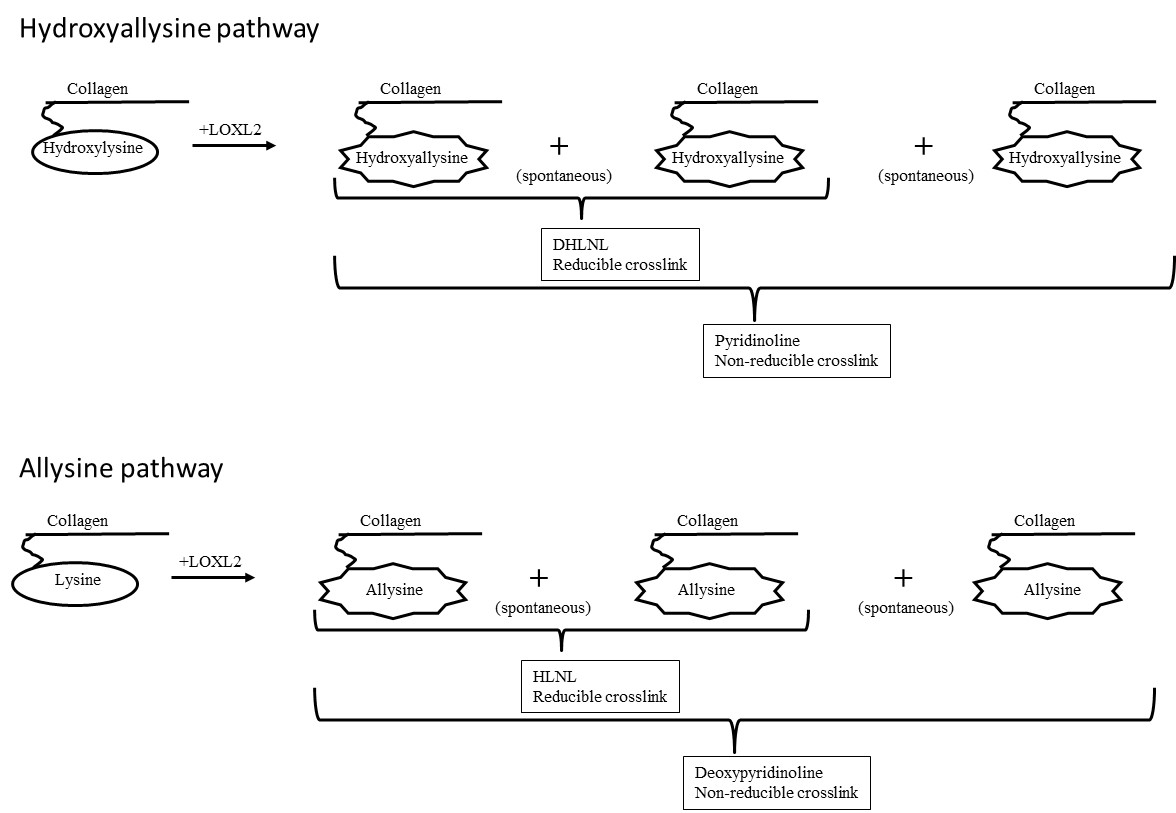


Figure 2

PXS-5153A compound chemical properties

Figure 3

Further experimental evidence in support of the postulated mechanism based inhibition include jump dilution experiments, in which a 100-fold dilution from 30xIC_50_ of the inhibitor led only to a small recovery in enzyme activity of PXS-5153A and almost full recovery of the corresponding analogue lacking the leaving group. Notably, when the jump dilution experiment was performed using LOXL1, enzymatic activity was fully recovered upon dilution.

PXS-5153A is a fast acting inhibitor, as judged by the time-dependent shift in the concentration response curves (main figure 1A). Fast inhibition is a desirable profile for a drug and an improvement over the LOXL2 inhibitor, PAT-1251 (racemate), which takes about 4 hours to achieve complete inhibition (Figure 3B).

(A) Irreversibility of inhibition of enzyme activity. Enzyme was preincubated 30 x IC_50_ of PXS-5153A, enzyme/inhibitor complex was diluted 100x and remaining activity assessed by AR/HRP assay. (B) Concentration and time dependent inhibition of LOXL2 activity by the racemate of PAT-125, using putrescine as a substrate.

Figure 4

The rate of *in vivo* crosslink formation and inhibition by PXS-5153A was compared with hydroxyproline formation.

(A) DHLNL. (B) HLNL. (C) PYD. (D) DPD. Data are presented as mean values ± SEM for n = 6 Sham and n = 14-15 CCl_4_. Data are compared using Student Two tailed t test. *p < 0.05, **p < 0.01, ***p < 0.001, ****p < 0.0001 compared to the CCl_4_ group; ####p < 0.0001 compared to the sham control.

Figure 5

Scatter plots show correlation between immature or mature crosslinks and CCl_4_-induced liver fibrosis. Mature crosslink density on the horizontal axis versus (A) ALT (B) AST (C) % fibrotic area. Immature crosslink density on the horizontal axis versus (D) ALT (E) AST (F) % fibrotic area.

Figure 6

Mature and immature crosslink density correlates with CCl_4_ induced liver fibrosis. (A) ALT vs DHLNL. (B) AST vs DHLNL. (C) % fibrotic area vs DHLNL. (D) ALT vs PYD. (E) AST vs PYD. (F) % fibrotic area vs PYD. (G) ALT vs HLNL. (H) AST vs HLNL. (I) % fibrotic area vs HLNL. (J) ALT vs DPD. (K) AST vs DPD. (L) % fibrotic area vs DPD.

Figure 7

Mature and immature crosslink density correlates with NASH liver fibrosis model. Individual crosslink scatter plots show correlation between DHLNL, PYD and liver fibrosis. (A) PYD vs % fibrotic area .(B) PYD vs NASH score. (C) DHLNL vs % fibrotic area. (D) DHLNL vs NASH score.

**Supporting information: Tables**

Table 1

Eurofins Hit Profiling Screen, was performed to evaluate the off -target profile of PXS-5153A. Biochemical assay results are presented as the percent inhibition of specific binding or activity.

| **Assay Name** | **% inhibition** |
| --- | --- |
| Adenosine A1 | 9 |
| Adenosine A2A | 0 |
| Adrenergic α1A | 20 |
| Adrenergic α1B | 6 |
| Adrenergic α2A | 97 |
| Adrenergic β1 | 3 |
| Adrenergic β2 | -5 |
| Calcium Channel L-Type, Dihydropyridine | 80 |
| Cannabinoid CB1 | 17 |
| Dopamine D1 | 6 |
| Dopamine D2S | 10 |
| GABAA, Flunitrazepam, Central | 26 |
| GABAA, Muscimol, Central | -7 |
| Glutamate, NMDA, Phencyclidine | 3 |
| Histamine H1 | 23 |
| Imidazoline I2, Central | 57 |
| Muscarinic M2 | 58 |
| Muscarinic M3 | 9 |
| Nicotinic Acetylcholine | 16 |
| Nicotinic Acetylcholine α1, Bungarotoxin | 2 |
| Opiate μ (OP3, MOP) | 11 |
| Phorbol Ester | 2 |
| Potassium Channel [KATP] | 8 |
| Potassium Channel hERG | 47 |
| Prostanoid EP4 370768 | 11 |
| Rolipram | 28 |
| Serotonin (5-Hydroxytryptamine) 5-HT2B | 33 |
| Sigma σ1 | 8 |
| Sodium Channel, Site 2 | 57 |
| Transporter, Norepinephrine (NET) | 15 |

**Table 2**

The importance of the leaving group of PXS-5153A was underscored by the efficacy seen by the corresponding analogue lacking the leaving group.

| **Assay** | **IC_50_ (nM)** |
| --- | --- |
| Recombinant human LOXL2 | 510 |
| Recombinant human LOXL3 | 970 |
|  |  |

**Table 3**

Pharmacokinetic profile of PXS-5153A in rodents.

|  | **Rat** | **Mouse** |
| --- | --- | --- |
| **Oral dose** | **10 mg/kg** | **5 mg/kg** |
| Bioavailability (%) | 10 | 40 |
| Apparent concentration at curve maximum C_max_ (ng/ml) | 33 | 113 |
| Time of maximal concentration T_max_ (hour) | 0.625 | 1 |
| Clearance CL (ml/kg/min) | 2029 | 593 |
|  |  |  |
| **Intravenous dose** | **5 mg/kg** | **5 mg/kg** |
| Apparent concentration at curve maximum C_max_ (gg/ml) | 1541 | 965 |
| Time of maximal concentration T_max_ (hour) | 0.03 | 0.08 |
| Clearance CL (ml/kg/min) | 180 | 673 |
| Apparent half-life, t_1/2_ (h) | 1.3 | 1.6 |
|  |  |  |

**Supporting information: References**

1. **Zhou M, Diwu Z, Panchuk-Voloshina N*, et al.*** A stable nonfluorescent derivative of resorufin for the fluorometric determination of trace hydrogen peroxide: applications in detecting the activity of phagocyte NADPH oxidase and other oxidases. *Analytical biochemistry*. 1997; 253: 162-8.

2. **Schilter HC, Collison A, Russo RC*, et al.*** Effects of an anti-inflammatory VAP-1/SSAO inhibitor, PXS-4728A, on pulmonary neutrophil migration. *Respiratory research*. 2015; 16: 42.

3. **Copeland RA, Basavapathruni A, Moyer M*, et al.*** Impact of enzyme concentration and residence time on apparent activity recovery in jump dilution analysis. *Analytical biochemistry*. 2011; 416: 206-10.
